# The combined evaluation of preoperative serum CEA and postoperative tissue CEA as a prognostic factor in stages 0–IV colorectal cancer: a retrospective cohort study

**DOI:** 10.3389/fmed.2024.1447041

**Published:** 2025-01-03

**Authors:** Guojun Tong, Hui Li, Yan Shen, Zhenhua Tan, Hai Qian

**Affiliations:** ^1^Department of Colorectal Surgery, Huzhou Central Hospital, The Affiliated Central Hospital of Huzhou University, Zhejiang, China; ^2^Department of Central Laboratory, Huzhou Central Hospital, The Affiliated Central Hospital of Huzhou University, Zhejiang, China; ^3^Department of General Surgery, Huzhou Central Hospital, The Affiliated Central Hospital of Huzhou University, Zhejiang, China

**Keywords:** CRC, prognosis, sCEA, tCEA, combined CEA, ROC, AUC, OS

## Abstract

**Background:**

The roles of preoperative serum carcinoembryonic antigen (sCEA) and postoperative tissue carcinoembryonic antigen (tCEA) have been extensively studied in isolation in colorectal cancer (CRC). However, the combined role of sCEA and tCEA remains inadequately described.

**Methods:**

A total of 1,757 retrospective cases of stage 0–IV CRC from January 2006 to January 2016 in our institution were included. Clinicopathological features and follow-up data were collected. Stage 0 was combined with stage I. sCEA levels were classified as normal or high (>10 ng/mL), while tCEA levels were categorised into three grades (+, ++, and +++). This resulted in six combined groups (2 × 3). ANOVA and cross-tabulation were employed to analyse continuous and categorical data, respectively. Univariate and multivariate analyses were conducted using Cox regression. All data were analysed using SPSS 27 and R 4.3.1.

**Results:**

Some clinicopathologic features differed significantly among the combined CEA test groups (all *p* < 0.05). The receiver operating characteristic (ROC) curves for sCEA, tCEA, and combined CEA exhibited significant differences in five-year OS with death as the input variable (all *p* < 0.05). The area under the curve (AUC) for combined CEA was the highest, indicating the value of this study. Cox regression analysis demonstrated that tumour location, T stage, differentiation, chemotherapy, TNM stage, tCEA, and combined CEA were significant in the univariate analysis; however, tCEA was not significant (*p* = 0.096) in the multivariate analysis among these seven variables. Five-year OS analysis revealed that sCEA, tCEA, and combined CEA were not significant in stages 0 & I–II (all *p* > 0.05) but were significant in stages III–IV (all *p* < 0.05), except for tCEA in stage IV (*p* = 0.24) as per K–M and univariate analysis. No significant difference was observed between sCEA and tCEA (*p* = 0.55, 0.095), whereas combined CEA demonstrated a significant difference (*p* < 0.001) in both univariate and multivariate analyses.

**Conclusion:**

sCEA, tCEA, and combined CEA exhibit prognostic roles in stages III–IV of CRC, with only combined CEA serving as an independent factor in these stages.

## 1 Introduction

Colorectal cancer (CRC) is one of the most prevalent cancers worldwide, posing a significant threat to human health and reducing patient survival. Colorectal adenocarcinoma accounts for approximately three-quarters of CRC cases (1). Despite decades of intensive research, the molecular mechanisms underlying this disease remain elusive. It is widely accepted that CRC is a genetic disease resulting from accumulated mutations in tumour suppressor genes and oncogenes, a phenomenon referred to as genomic instability (2). Tumour markers not only indicate the presence of cancer but also provide crucial information regarding treatment response and progression (3). Carcinoembryonic antigen (CEA) levels are often elevated in CRC patients and are associated with a worse prognosis (4, 5). Serum CEA (sCEA) is widely used as a tumour marker in CRC (6), with sCEA levels correlating with tumour stage and metastasis (7). Our previous study demonstrated that high sCEA levels are associated with poor prognosis in stage III CRC (8). Ma et al. (9) indicated that sCEA levels ≥5 ng/mL are indicative of poor prognosis in CRC. Levels of sCEA are significantly higher in the gastrointestinal tumour group compared with healthy individuals (7). Elevated preoperative sCEA concentrations, defined as >5 ng/mL or more than twice the normal cut-off value, are significantly associated with poorer overall and higher cancer-specific mortality in CRC patients (10). sCEA is a clinically established serum biomarker for CRC diagnosis (11). Preoperatively elevated sCEA levels are reliable predictors of high-risk postoperative recurrence in CRC and, when combined with TNM stage, precisely identify postoperative recurrence in stages I–III CRC patients, as well as the benefit of adjuvant chemotherapy for stage II CRC patients (12). Tissue CEA (tCEA) expression can be assessed immunohistochemically in colorectal mucosa and tumour tissues. tCEA is rarely expressed in normal colorectal mucosa but is consistently found in colorectal neoplasms, exhibiting varying expression patterns and intensities (10, 13, 14). Aldilaijan et al. (10) found that tCEA expression intensity and pattern correlate significantly with preoperative sCEA levels. In their study involving 7,412 patients, only 100 (1.3%) showed inverse relationships between tCEA expression intensities and preoperative sCEA levels. Low tCEA expression intensity in patients with high preoperative sCEA levels may be explained by factors unrelated to malignancy, including the wide range of normal preoperative sCEA concentrations among healthy individuals, the effects of age and benign conditions, the high variability of liver metabolic rates, and the long half-life of glycoproteins. Our previous research indicated that higher tCEA levels are associated with a worse prognosis in stages I–III CRC (8). However, the prognostic value of tCEA in CRC has been rarely reported. Polivka et al. (15) suggested that optimal prognostic value could be achieved by combining circulating cell-free tumour DNA (ctDNA) with the tumour marker CEA. The combination of CEA, carbohydrate antigen 19-9 (CA19-9), and carbohydrate antigen 24-2 (CA24-2) demonstrated the highest sensitivity and specificity for CRC diagnosis (16). Preoperative serum CA724 may serve as a potential prognostic factor for CRC patients with normal serum CEA levels (17). However, Kemper et al. (18) pointed out that only CEA was an independent prognostic factor for survival according to multivariate Cox regression analysis. Another study demonstrated that serum carbohydrate antigen 19-9 (CA19-9), recurrence-free survival (RFS), and OS were evaluated in patients with or without elevated sCEA (19). Thus far, the prognostic value of sCEA and tCEA remains controversial. The aim of this study is to assess the prognostic values of combining sCEA and tCEA in CRC.

In this study, we utilised the combined CEA factor to explore the prognostic values of CRC based on different levels of sCEA [normal (<10 ng/mL), high (≥10 ng/mL)] and varying expression levels of tCEA (+, ++, and +++), as classified in our previous research (8). Therefore, combined CEA was categorised into six grades (2 × 3). Prior to conducting the study, we evaluated the value of combined CEA using the receiver operating characteristic (ROC) curve, which demonstrated significant differences in five-year OS with death as the input variable for sCEA, tCEA, and combined CEA (all *p* < 0.05). The area under the curve (AUC) for combined CEA was the highest, indicating its value and necessity compared with analysing only sCEA and tCEA for the prognostic role in CRC.

## 2 Materials and methods

### 2.1 Patients

A total of 2,540 CRC patients were identified in the Colorectal Surgery Department of Huzhou Central Hospital, China, from January 2006 to January 2016. Of these, 783 cases were excluded for various reasons, including lack of surgery, missing clinicopathological data, missing follow-up data, and deaths not related to primary tumours. Ultimately, 1,757 retrospective cases were included in this study. The case collection routine adhered to our previous literature, utilising the same dataset (20).

The inclusion criteria were as follows: patients diagnosed with CRC through colonoscopy, computed tomography (CT), and pathological tests conducted either in our hospital or externally; no preoperative adjuvant treatment; surgery performed in our department; normal lymph node dissection, indicating that ≥12 lymph nodes were detected (though a small number of samples with only 10–11 lymph nodes were included in this article); CRC-related death as a termination event; postoperative routine immunohistochemical (IHC) analysis and pathological examination for tCEA; and postoperative chemotherapy determined by the National Comprehensive Cancer Network (NCCN, version 2006) guidelines. The TNM stage was determined using the American Joint Committee on Cancer (AJCC-8) guidelines after surgery.

The exclusion criteria were as follows: CRC patients with severe heart, brain, liver, or lung diseases that precluded surgery; non-CRC factors leading to patient death; missing follow-up or clinicopathological data; and patients who had undergone preoperative neoadjuvant chemotherapy and radiotherapy. In accordance with previous literature, stage 0 was combined with stage I, referred to as stages 0 & I in this study (21).

### 2.2 Follow-up

Patients were followed up every 3 months during the first year after primary CRC surgery, then every 6 months in the second year, and annually for the remaining 3 years, totalling 5 years. All follow-up data were obtained from our records, either by phone or through the inpatient electronic medical record system (Haitai Software Version 3.0, Nanjing). Survival time was calculated from the date of primary surgery to the date of death or the end of the follow-up period, which was at least 5 years. If the survival time exceeded 60 months, it was defined as 60 months. Death due to the primary tumour or tumour-related disease was defined as a positive event, while other causes were considered censoring events. Thus, in this study, only OS was analysed.

### 2.3 Detection of preoperative serum CEA

Venous blood was drawn from each patient before surgery and analysed using a kit from Shanghai Yu-ping Biotechnology Company (Shanghai, China), employing a double antibody one-step enzyme-linked immunosorbent assay (ELISA). Preparation of serum samples: use test tubes without heat sources and endotoxins, avoid any cell irritation during the operation, collect blood, centrifuge at 3,000 rpm, and then take blood serum. Reagent preparation: dilute distilled water at a ratio of 1:20, that is, add 19 parts of distilled water to one part of 20× washing buffer. Automatic plate washing method: inject 350 μL of washing solution into each well, soak for 1 min, and wash the plate 5 times. Operation steps: the experimenters sequentially added the sample, standard, and horseradish peroxidase (HRP)-labelled detection antibody to microcells precoated with the CEA capture antibody. After an incubation period, the wells were washed, and absorbance (optical density value) was measured at a wavelength of 450 nm using a microplate reader to calculate the sample concentration (the normal reference value is 0–10 ng/mL). An sCEA level >10 ng/mL is considered high, while ≤10 ng/mL is regarded as normal.

### 2.4 tCEA immunohistochemistry

**Figure 1:**
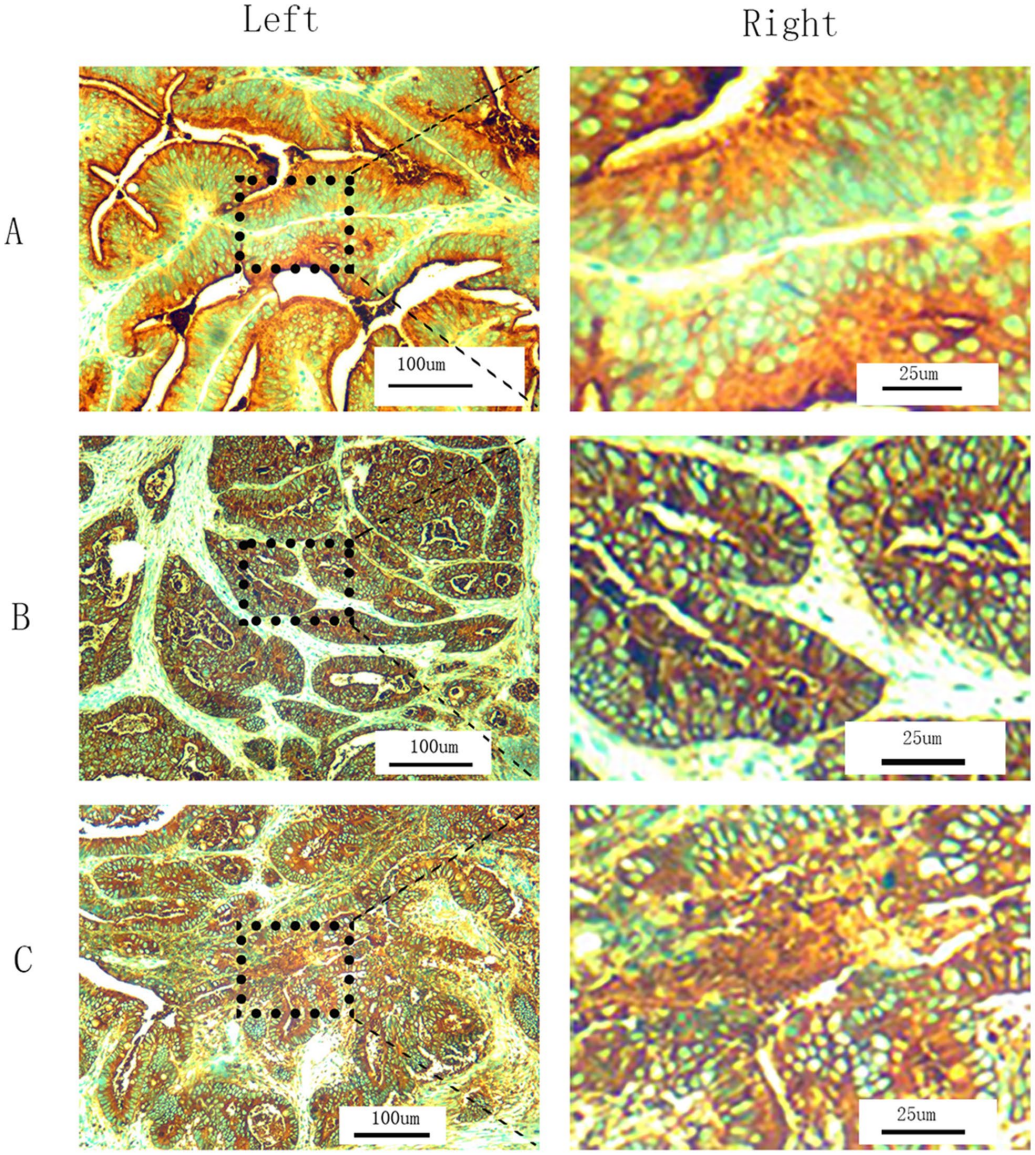
T-CEA immunohistochemistry. **(A)** Staining level is +. **(B)** Staining level is ++. **(C)** Staining level is +++. (Left) 200× magnification under microscope (original images). (Right) 800× magnification by Photoshop using the specific regions of original images.

Our hospital uses immunohistochemistry (IHC) to detect tCEA for pathological examination of CRC specimens. Tumor specimens fixed in formalin and embedded in paraffin were sliced into 5 μm thick sections, deparaffinized to water, and subjected to EnVision two-step immunohistochemistry system. The original anti CEA antibody (clone number COL-1, zm-0061, Jinqiao Company, Beijing, China) diluted 1:50 was used; conducted 15 min EDTA thermal repair, incubated in a 37°C oven for 30 min, and then incubated with secondary antibody PV8000 for 15 min at 37 ° C. DAB staining was performed, and hematoxylin counter-stain was used to dehydrate and seal the slides. Microscopic examination confirmed the percentage of CEA positive stained cytoplasm cells. All slides were independently analyzed by two trained pathologists. In case of any disagreement, a third pathologist can be consulted to reduce evaluation bias to a certain extent. All slides were observed under 200× magnification to determine cell density (+, ++, and +++) and the corresponding proportions (≤25, >25%, ≤50, and >50%) of stained cells in different regions. The tCEA images shown in Figures 1A–C included 200× magnification images for clarity (Figure 1, left) and 800× magnification images for enhanced detail, processed using Photoshop (Version 2020, Figure 1, right).

### 2.5 Combined CEA classification

According to previous classifications, sCEA was divided into two grades, and tCEA into three grades. Combined CEA was therefore classified into six grades: sCEA normal & tCEA+, sCEA normal & tCEA++, sCEA normal & tCEA+++, sCEA high & tCEA+, sCEA high & tCEA++, and sCEA high & tCEA+++. All data were analysed within these six groups.

### 2.6 Receiver operating characteristic curve analysis

ROC analysis was performed for sCEA, tCEA, and combined CEA using the death event of five-year OS as the input parameter to determine the necessity and priority of the combined CEA classification in this study. AUC analysis confirmed the value of this approach.

### 2.7 Statistical analysis

All clinicopathological features were analysed using SPSS 27. ANOVA and crosstab methods were employed to analyse continuous and categorical variables, respectively. Meadian and interquartile range (IQR) were carried out using Exploring by SPSS27. Comparisons between combined CEA groups were performed using the *F* and *χ*^2^ tests. Kaplan–Meier and log-rank tests were utilised for survival analysis between sCEA, tCEA, and combined CEA groups. Cox regression analysis was applied for univariate and multivariate analyses. MultiVarTimeRoc and five-year OS survival curves with numbers at risk were generated using R software (version 4.3.1) with the “ggplot2,” “survival,” “survminer,” and “timeROC” packages.

## 3 Results

### 3.1 sCEA and tCEA distributions

The high sCEA level was observed in 51.6% (906/1,757) of patients, while the normal sCEA level was recorded in 48.4% (861/1,757). The percentages of tCEA expression were 34.2% (601/1,757) for +, 44.6% (784/1,757) for ++, and 21.2% (372/1,757) for +++.

### 3.2 Clinicopathological features by combined CEA

**Figure 2:**
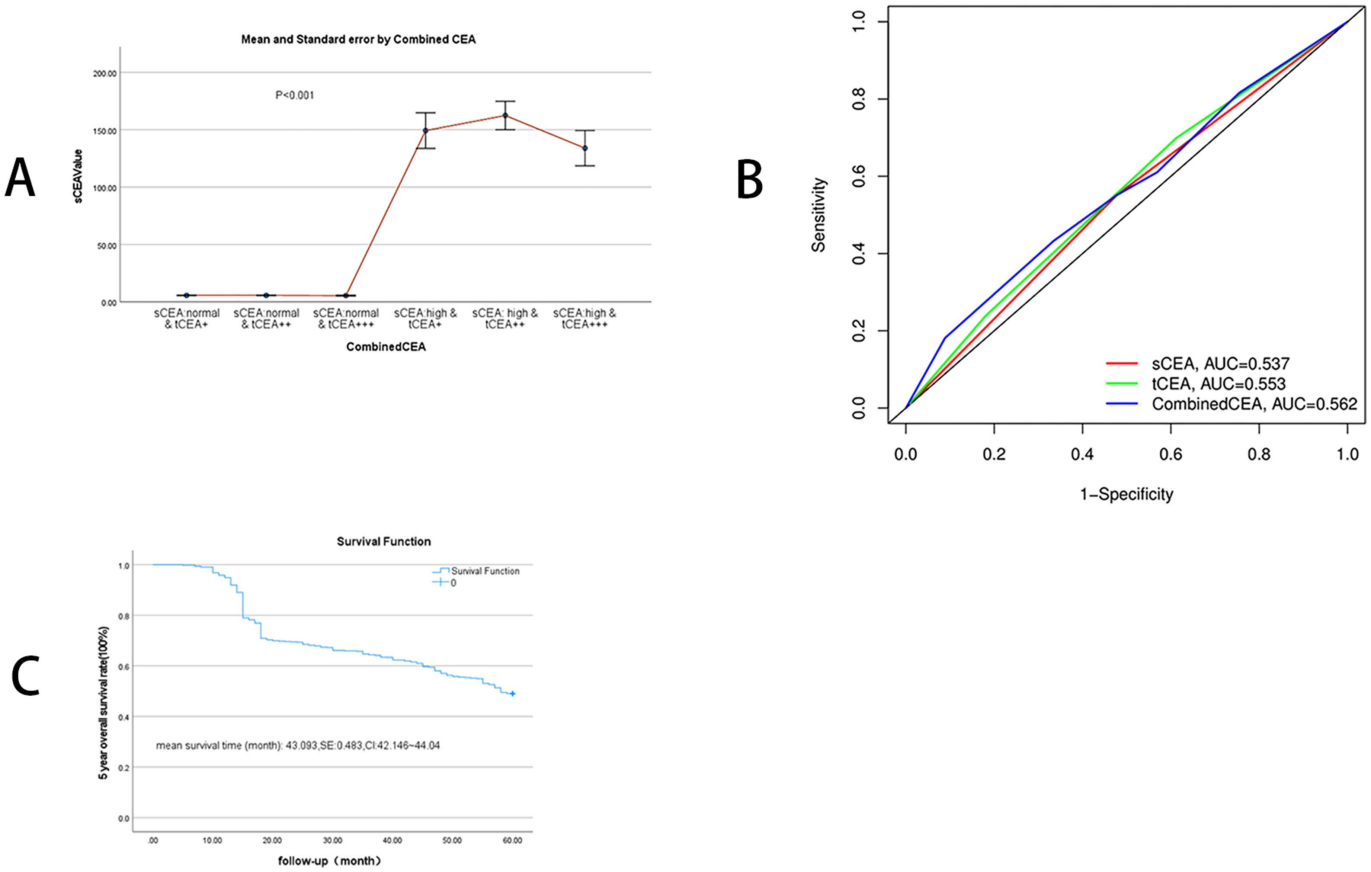
SCEA value distribution between combined CEA groups and receiver operating characteristic curve (ROC) and 5-year OS. **(A)** SCEA value distributions: mean and standard error, *p* < 0.001. **(B)** ROC analysis: sCEA and tCEA and combined CEA are as follows: AUC = 0.537, CI = 0.512–0.566, *p* = 0.004; AUC = 0.553, CI = 0.531–0.584, *p* < 0.001; AUC = 0.562, CI = 0.540–0.593, *p* < 0.001. **(C)** 5-year OS: mean survival time of 5-year OS in this study is 43.093 (months), SE = 0.483, and 95% CI = 42.146–44.040.

The percentages of combined CEA classifications are as follows: 21.2% (372/1,757) for sCEA normal & tCEA+, 19.5% (343/1,757) for sCEA normal & tCEA++, 7.7% (136/1,757) for sCEA normal & tCEA+++, 13.1% (230/1,757) for sCEA high & tCEA+, 24.9% (438/1,757) for sCEA high & tCEA++, and 13.5% (238/1,757) for sCEA high & tCEA+++. Significant differences were observed in gender between combined CEA groups (*F* = 12.22, *p* = 0.032). Age differences between groups were also significant (*χ*^2^ = 5.37, *p* < 0.001). Significant differences among combined CEA groups were noted for various parameters: *F* = 202.11, *p* < 0.001; *F* = 452.82, *p* < 0.001; *F* = 22.25, *p* < 0.001; *F* = 160.92, *p* < 0.001; *F* = 58.60, *p* < 0.001. Continuous parameters, expressed as median and interquartile range (IQR), also exhibited significant differences between groups: age (*χ*^2^ = 5.37, *p* < 0.001), tumour size (cm) (*χ*^2^ = 18.60, *p* < 0.001), blood loss (mL) (*χ*^2^ = 3.51, *p* = 0.004), sCEA value (ng/mL) (*χ*^2^ = 231.65, *p* < 0.001, Figure 2A), harvested lymph nodes (number) (*χ*^2^ = 6.83, *p* < 0.001), and metastatic positive lymph nodes (number) (*χ*^2^ = 22.17, *p* < 0.001). A positive relationship was found between sCEA and tCEA (Pearson correlation = 0.194, *p* < 0.001). Counting data were presented as numbers and percentages, while continuous data were presented as medians and IQR. Detailed information is provided in Table 1.

**Table 1:** Clinicopathological features by combined CEA (*n*, %; median, IQR).

Variables	sCEA: normal & tCEA+	sCEA: normal & tCEA++	sCEA: normal & tCEA+++	sCEA: high & tCEA+	sCEA: high & tCEA++	sCEA: high & tCEA+++	*F* or *χ*^2^ test	*p*
Gender							12.22	0.032^*^
Male	186 (10.6)	165 (9.4)	74 (4.2)	111 (6.3)	209 (11.9)	143 (8.1)		
Female	186 (10.6)	178 (10.1)	62 (3.5)	119 (6.8)	229 (13.0)	95 (5.4)		
Age (year)	67 (16)	65 (15)	67 (21.75)	65 (19.5)	67 (21)	67 (15)	5.37	<0.001^***^
Location							202.11	<0.001^***^
Ileocecum	41 (2.3)	33 (1.9)	18 (1.0)	11 (0.6)	7 (0.4)	37 (2.1)		
Right colon	30 (1.7)	76 (4.3)	8 (0.5)	12 (0.7)	38 (2.2)	8 (0.5)		
Transverse colon	66 (3.8)	39 (2.2)	21 (1.2)	42 (2.4)	86 (4.9)	23 (1.3)		
Left colon	84 (4.8)	32 (1.8)	36 (2.0)	38 (2.2)	87 (5.0)	47 (2.7)		
Sigmoid colon	35 (2.0)	45 (2.6)	12 (0.7)	31 (1.8)	18 (1.0)	26 (1.5)		
Rectum	116 (6.6)	118 (6.7)	41 (2.3)	96 (5.5)	202 (11.5)	97 (5.5)		
Tumor size (cm)	3.7 (1.1)	4.1 (1)	3.6 (1)	3.5 (1.2)	3.5 (0.9)	4.1 (1.33)	18.60	<0.001^***^
Blood loss (mL)	180 (110)	160 (150)	180 (115)	180 (160)	180 (110)	160 (52.5)	3.51	0.004^**^
SCEA value (ng/mL)	5.5 (3.7)	5.5 (3.7)	5.5 (3.7)	125 (208)	125 (198)	79 (202)	231.65	<0.001^***^
T stage							452.82	<0.001^a^^,***^
Tis	9 (0.5)	3 (0.2)	3 (0.2)	1 (0.1)	0 (0)	0 (0)		
T1	23 (1.3)	37 (2.1)	5 (0.3)	21 (1.2)	20 (1.1)	7 (0.4)		
T2	106 (6.0)	26 (1.5)	30 (1.7)	24 (1.4)	91 (5.2)	37 (2.1)		
T3	53 (3.0)	157 (8.9)	73 (4.2)	117 (6.7)	177 (10.1)	53 (3.0)		
T4a	23 (1.3)	29 (1.7)	24 (1.4)	66 (3.8)	92 (5.2)	45 (2.6)		
T4b	158 (9.0)	91 (5.2)	1 (0.1)	1 (0.1)	58 (3.3)	96 (5.5)		
Differentiation							22.25	<0.001^***^
Well	26 (1.5)	66 (3.8)	19 (1.1)	31 (1.8)	59 (3.4)	24 (1.4)		
Moderate	234 (13.3)	251 (14.3)	75 (4.3)	136 (7.7)	350 (19.9)	90 (5.1)		
Poor or undifferentiation	112 (6.4)	26 (1.5)	42 (2.4)	63 (3.6)	29 (1.7)	124 (7.1)		
Harvested lymph node (No.)	14 (3)	13 (3)	14 (3)	14 (2)	14 (2)	14 (3)	6.83	<0.001^***^
Positive lymph node (No.)	2 (2)	2 (6)	0 (2)	2 (6)	3 (5)	2 (5)	22.17	<0.001^***^
Chemotherapy							22.25	<0.001^***^
Yes	319 (18.2)	295 (16.8)	117 (6.7)	200 (11.4)	402 (22.9)	227 (12.9)		
No	53 (3.0)	48 (2.7)	19 (1.1)	30 (1.7)	36 (2.0)	11 (0.6)		
TNM stage							160.92	<0.001^a^^,***^
0 & I	42 (2.4)	30 (1.7)	12 (0.7)	26 (1.5)	28 (1.6)	9 (0.5)		
II	26 (1.5)	49 (2.8)	42 (2.4)	28 (1.6)	47 (2.7)	33 (1.9)		
III	170 (9.7)	133 (7.6)	82 (4.7)	136 (7.7)	209 (11.9)	145 (8.3)		
IV	134 (7.6)	131 (7.5)	0 (0)	40 (2.3)	154 (8.8)	51 (2.9)		
Complication							58.60	<0.001^***^
No	357 (20.3)	284 (16.2)	121 (6.9)	214 (12.2)	421 (12.2)	215 (12.2)		
Yes	15 (0.9)	59 (3.4)	15 (0.9)	16 (0.9)	17 (1.0)	23 (1.3)		

No., numbers; IQR, interquartile range; ^*^indicates *p* < 0.05; ^**^indicates *p* < 0.01; ^***^indicates *p* < 0.001.

a Respected values <5 and using exact test.

### 3.3 Receiver operating characteristic curve analysis

ROC analysis was conducted for sCEA, tCEA, and combined CEA using the death event of five-year OS as the input parameter. AUC analysis confirmed the value of this study, yielding the following results: sCEA (AUC = 0.537, CI = 0.512–0.566, *p* = 0.004), tCEA (AUC = 0.553, CI = 0.531–0.584, *p* < 0.001), and combined CEA (AUC = 0.562, CI = 0.540–0.593, *p* < 0.001). The AUC for combined CEA was the highest among the three variables, indicating its value as a significant factor in this study (Figure 2B). In this analysis, the binary variable was death within 5 years, while the other variable was censoring. The mean survival time for five-year OS in this study was 43.093 months, SE = 0.483, and 95% CI = 42.146–44.040 (Figure 2C).

### 3.4 Five-year OS analysis by sCEA, tCEA, and combined CEA for stages 0 & I–IV CRC of AJCC-8

**Figure 3:**
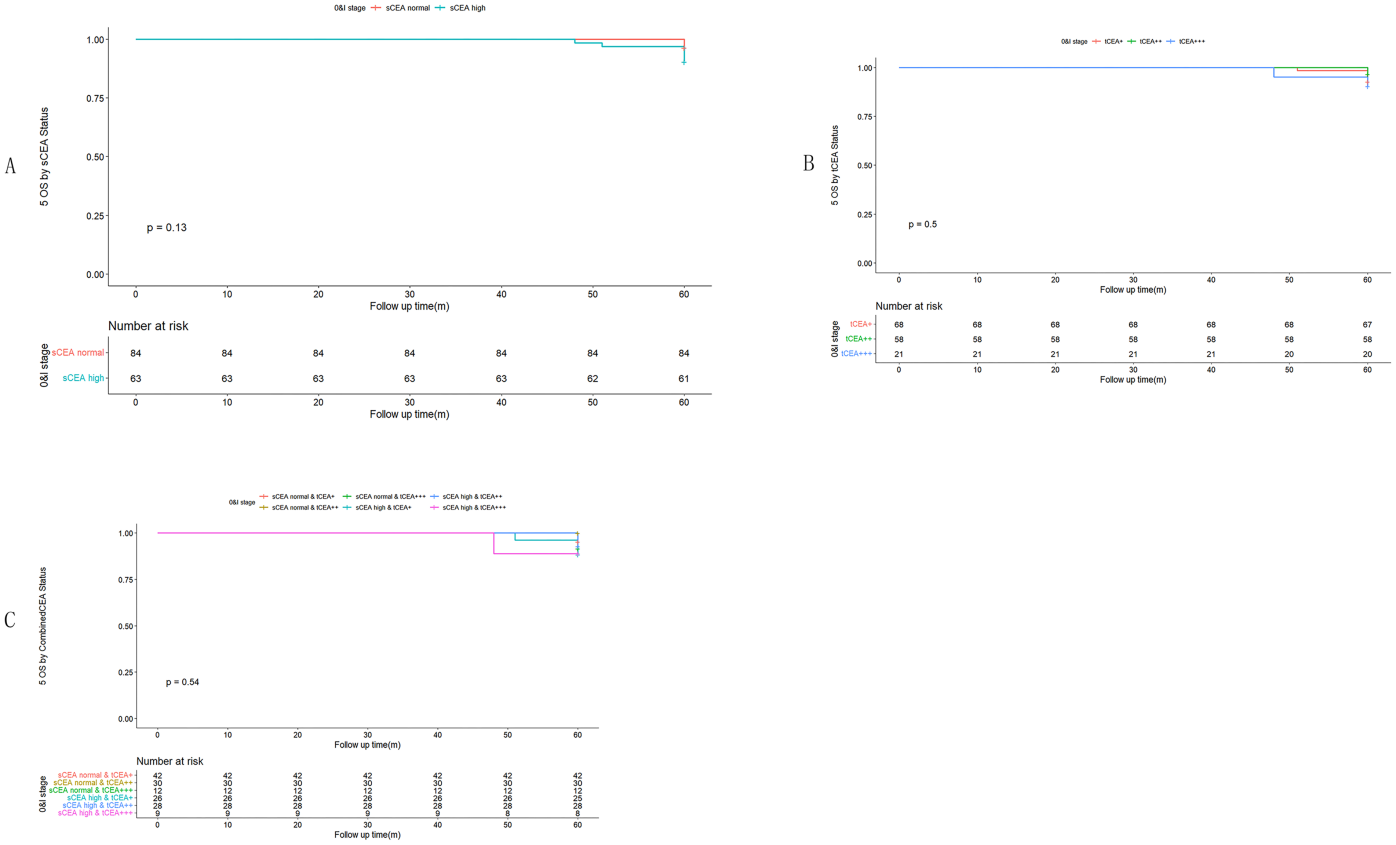
Comparisons for subgroups of sCEA, tCEA, and combined CEA in 0 & I stages. **(A)** Comparison for subgroups of sCEA (*p* = 0.13). **(B)** Comparison for subgroups of tCEA (*p* = 0.5). **(C)** Comparison for subgroups of combined CEA (*p* = 0.54).

**Figure 4:**
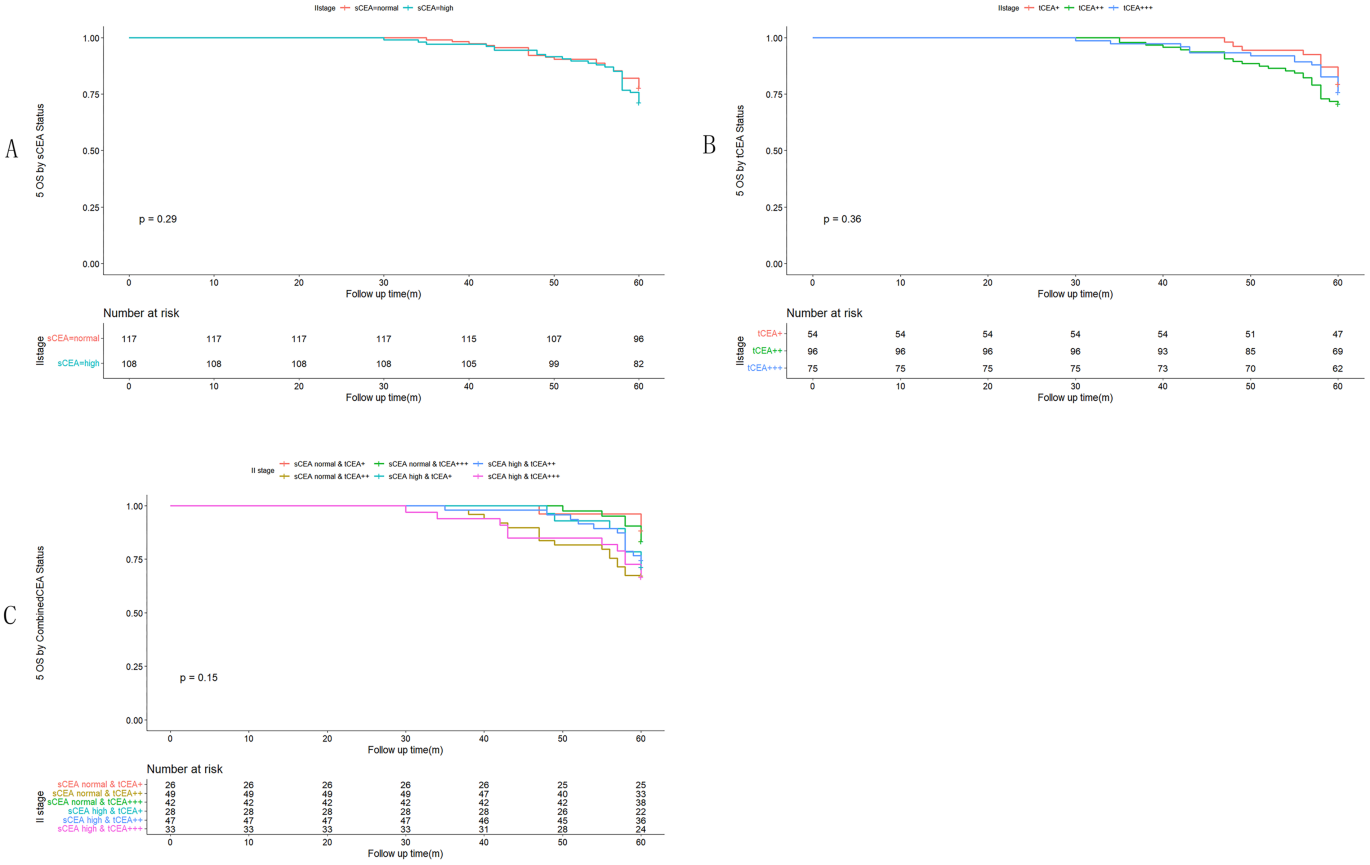
Comparisons for subgroups of sCEA, tCEA, and combined CEA in II stage. **(A)** Comparison for subgroups of sCEA (*p* = 0.29). **(B)** Comparison for subgroups of tCEA (*p* = 0.36). **(C)** Comparison for subgroups of combined CEA (*p* = 0.15).

**Figure 5:**
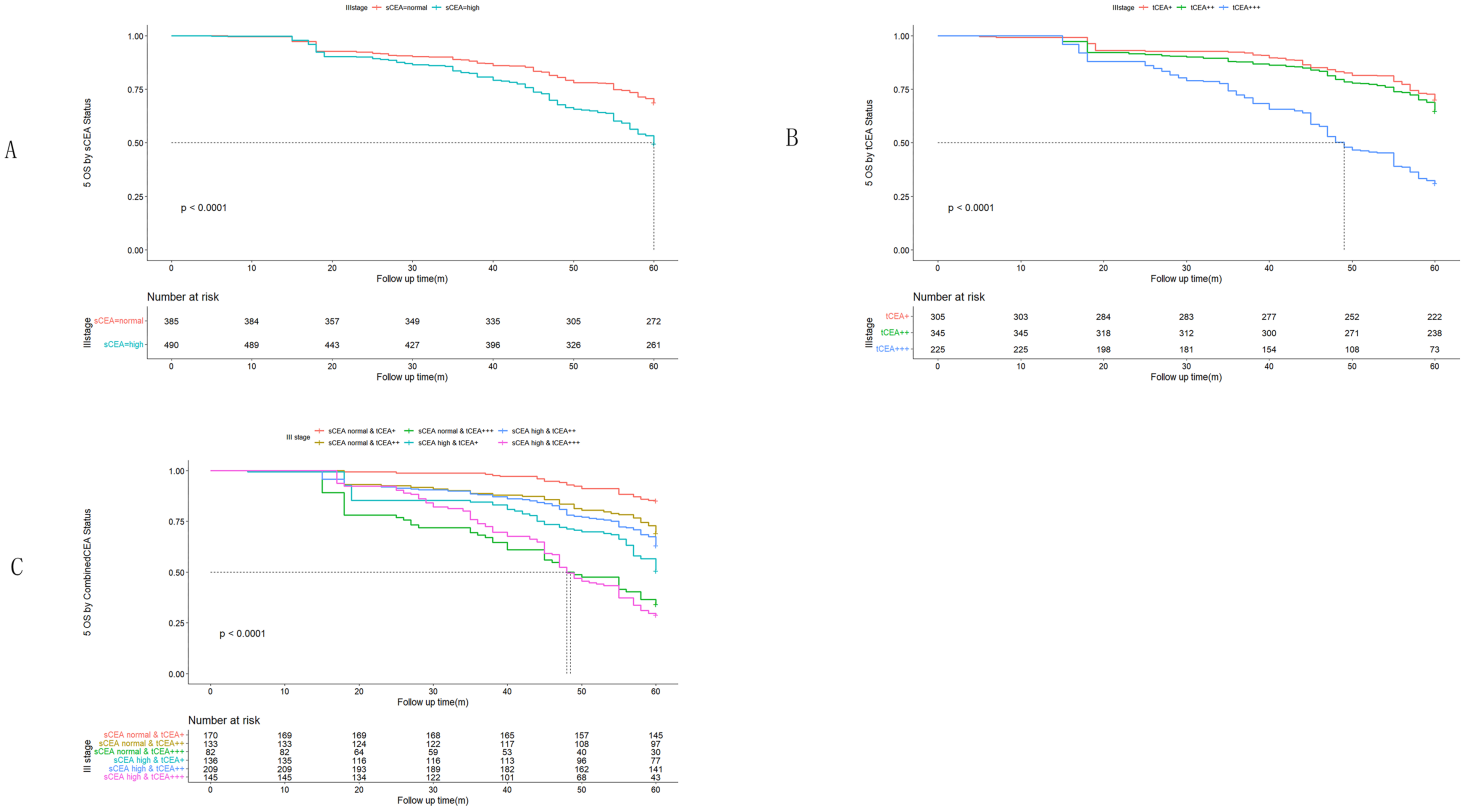
Comparisons for subgroups of sCEA, tCEA, and combined CEA in III stage. **(A)** Comparison for subgroups of sCEA (*p* < 0.001). **(B)** Comparison for subgroups of tCEA (*p* < 0.001). **(C)** Comparison for subgroups of combined CEA (*p* < 0.001).

**Figure 6:**
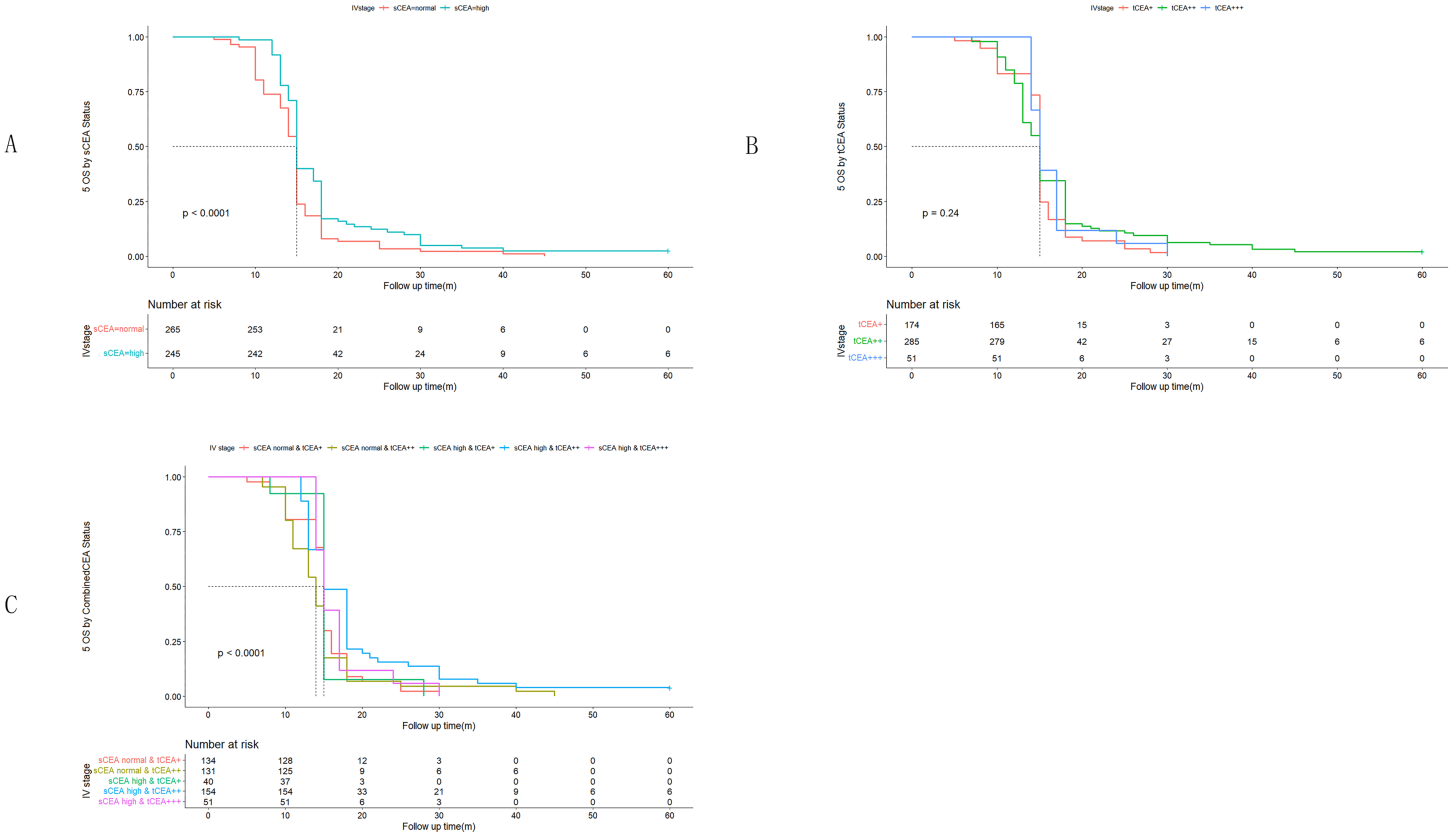
Comparisons for subgroups of sCEA, tCEA, and combined CEA in IV stage. **(A)** Comparison for subgroups of sCEA (*p* < 0.001). **(B)** Comparison for subgroups of tCEA (*p* = 0.24). **(C)** Comparison for subgroups of combined CEA (*p* < 0.001). In subgroup of sCEA normal & tCEA+++, there is no survival data.

In accordance with the study by Zhang et al. (21), we combined stage 0 with stage I due to insufficient data in some combined CEA groups, thus utilising the combined stages 0 & I. Five-year OS and numbers at risk were calculated for each stage of AJCC-8. In stages 0 & I, there were no significant differences in sCEA, tCEA, and combined CEA groups (*p* = 0.13, 0.50, 0.54, respectively; Figure 3). In stage II, no significant differences were noted in sCEA, tCEA, and combined CEA groups (*p* = 0.29, 0.36, 0.15, respectively; Figure 4). In stage III, all three classification methods demonstrated significant differences (all *p* < 0.001; Figure 5). In stage IV, significant differences were observed in sCEA and combined CEA (both *p* < 0.001), but not in tCEA (*p* = 0.24). In this stage, the group of sCEA normal & tCEA+++ was absent in combined CEA (Figure 6).

### 3.5 Univariate analysis by Cox regression for clinicopathological features

In the univariate analysis, there were no significant differences in age (*p* = 0.926), sCEA, and complications (*p* = 0.55, 0.85), indicating that age, sCEA, and complications are not prognostic factors for CRC in this study. However, significant differences were observed in tumour location, T stage, differentiation, chemotherapy, TNM stages, tCEA, and combined CEA (tCEA *p* = 0.002; all others *p* < 0.001). Numbers, hazard ratios (HR), mean survival times, 95% confidence intervals (CIs), five-year OS (%), and *p* values are detailed in Table 2.

**Table 2:** Univariate analysis of prognosis for colorectal cancer.

Factor	*N*	Hazard ratio (HR)	Mean and 95% CI for survival time (60 months)	5-year OS (%)	*p*-value
Gender					0.296
M	888	Ref.	42.78 (41.45–44.09)	47.5	
F	869	1.072	43.42 (42.06–44.78)	50.5	
Location					<0.001^***^
Ileocecum	147	Ref.	36.93 (33.42–40.49)	38.1	
Right colon	172	1.488	35.63 (32.42–38.85)	37.8	
Transverse colon	277	1.521	47.40 (45.24–49.56)	57.0	
Left colon	324	0.777	43.28 (41.13–45.44)	48.5	
Sigmoid colon	167	1.019	45.99 (42.95–49.03)	57.5	
Rectum	670	0.815	43.77 (42.27–45.27)	49.1	
T stage					<0.001^***^
Tis	16	Ref.	60 (60–60)	93.8	
T1	113	0.064	57.97 (56.93–59.02)	81.4	
T2	314	0.197	42.29 (40.04–44.53)	46.8	
T3	630	0.759	40.37 (38.75–41.99)	39.8	
T4a	279	0.911	52.96 (51.37–54.54)	69.9	
T4b	405	0.345	36.34 (34.26–38.42)	39.8	
Differentiation					<0.001^***^
Well	225	Ref.	52.88 (50.77–64.98)	76.9	
Moderate	1,136	0.159	45.27 (44.11–46.43)	56.2	
Poor or undifferentiation	396	0.355	31.30 (29.53–33.08)	12.6	
Chemotherapy					<0.001^***^
Yes	1,560	Ref.	41.09 (40.07–42.11)	44.0	
No	197	6.78	58.98 (58.46–59.50)	88.3	
TNM stage					<0.001^***^
0 & I	147	Ref.	59.86 (59.61–60.00)		
II	225	0.003	58.08 (57.40–58.76)		
III	875	0.027	51.98 (51.08–52.88)		
IV	510	0.060	16.40 (15.76–17.04)		
Complication					0.85
No	1,612	Ref.	42.95 (41.96–43.94)	49	
Yes	145	1.023	44.70 (41.60–47.79)	49	
sCEA					0.55
Normal	851	Ref.	42.91 (41.49–44.33)	53.0	
High	906	0.879	43.27 (42.00–44.53)	45.3	
tCEA					0.002^**^
+	601	Ref.	44.30 (42.65–45.94)		
++	784	0.762	41.27 (39.8–42.75)		
+++	372	0.985	44.99 (43.22–46.76)		
Combined CEA					<0.001^***^
sCEA: normal & tCEA+	372	Ref.	42.92 (40.73–45.10)	57.0	
sCEA: normal & tCEA++	343	0.413	40.06 (37.74–42.37)	46.6	
sCEA: normal & tCEA+++	136	0.833	50.07 (47.43–52.72)	58.1	
sCEA: high & tCEA+	230	0.509	46.57 (44.14–48.99)	53.9	
sCEA: high & tCEA++	438	0.599	42.30 (40.40–44.21)	48.2	
sCEA: high & tCEA+++	238	0.742	41.85 (39.60–44.11)	31.5	

Classified clinicopathological data were analyzed for univariate regression. Using Kaplan–Meier to analyze number, mean, 95% CI survival time, and Cox regression (input) to analyze hazard ratio (HR), *p*-value. ^*^p < 0.05, ^**^p < 0.01, and ^***^p < 0.001.

### 3.6 Multivariate analysis by Cox regression for clinicopathological features

Seven parameters that demonstrated significant differences in the univariate analysis were further examined using multivariate analysis. The results indicated that chemotherapy and tCEA were not significantly different (*p* = 0.433, 0.096), while tumour location, T stage, differentiation, TNM stage, and combined CEA exhibited significant differences (all *p* < 0.001). This study establishes that only combined CEA serves as an independent prognostic factor for CRC, whereas sCEA and tCEA do not. Details of the analysis, including comparisons, wards, and *p*-values, are presented in Table 3.

**Table 3:** Multivariate analysis of prognosis for colorectal cancer.

Factor	HR	95% CI for HR	Ward	*p*
Location			29.05	<0.001^***^
Ileocecum	Ref.			
Right colon	0.850	0.623–1.159		
Transverse colon	0.577	0.427–0.779		
Left colon	0.722	0.547–0.954		
Sigmoid colon	0.480	0.341–0.675		
Rectum	0.821	0.636–1.061		
T stage			95.93	<0.001^***^
Tis	Ref.			
T1	0.174	0.015–1.986		
T2	0.280	0.025–3.118		
T3	0.565	0.050–6.399		
T4a	0.306	0.027–4.472		
T4b	0.220	0.020–2.470		
Differentiation			190.18	<0.001^***^
Well	Ref.			
Moderate	2.142	1.572–2.917		
Poor or undifferentiation	6.794	4.806–9.605		
Chemotherapy			0.61	0.433
Yes	Ref.			
No	0.779	0.417–1.454		
TNM stage			954.18	<0.001^***^
0 & I	Ref.			
II	4.789	1.062–21.604		
III	9.632	2.194–42.292		
IV	267.44	60.944–1173.58		
tCEA			4.68	0.096
+	Ref.			
++	4.831	0.664–35.174		
+++	1.635	0.142–18.789		
Combined CEA			32.67	<0.001^***^
sCEA: normal & tCEA+	Ref.			
sCEA: normal & tCEA++	0.475	0.065–3.488		
sCEA: normal & tCEA+++	1.176	0.100–13.824		
sCEA: high & tCEA+	1.242	0.921–1.674		
sCEA: high & tCEA++	0.266	0.036–1.958		
sCEA: high & tCEA+++	1.275	0.111–14.606		

Factors which have significance in univariate analysis were analyzed by multivariate regression analysis. Cox regression was used for multivariate analysis. ^*^p < 0.05, ^**^p < 0.01, and ^***^p < 0.001.

## 4 Discussion

sCEA is widely utilised both preoperatively and postoperatively in CRC management (22). A high expression level of sCEA is generally associated with poor prognosis, serving as a prognostic tumour marker. CEA, a glycoprotein first identified by Gold and Freedman in colon cancer tissues, has since been employed as a CRC tumour marker (23). The expression of sCEA correlates with CRC prognosis and is primarily used for disease monitoring and as an indicator of treatment response (24). In this study, 64.7% of patients (101/156) exhibited elevated sCEA levels (22). Specifically, high sCEA levels were observed in 51.6% of cases, while normal sCEA levels were recorded in 48.4%. The percentages of tCEA (+, ++, and +++) were 34.2, 44.6, and 21.2%, respectively. In CRC, CEA expression follows the disruption of normal tissue architecture and the loss of polarity in neoplastic cells, leading to its secretion into the bloodstream and a consequent rise in sCEA concentration (25). tCEA expression patterns can be classified as apicoluminal (AL), diffuse-cytoplasmic (DC), or a combination of both. The DC pattern, particularly at elevated expression levels, has been linked to tumour aggressiveness, including lymphovascular invasion (LVI) (10, 26). Given that sCEA levels lack sufficient sensitivity and specificity as a screening tool for CRC (27), this study was conducted. A recent study similar to ours (10) combined sCEA and tCEA into a new variable, termed combined CEA, for further analysis.

In this study, the percentages of sCEA normal & tCEA+ and sCEA high & tCEA++ were higher than those of other groups in the combined CEA classification. Factors such as gender, tumour location, T stage, differentiation, chemotherapy, TNM stage, complications, age, tumour size, blood loss, harvested lymph nodes, and metastatic positive lymph nodes are associated with this new variable, suggesting its utility in analysing clinicopathological features. Although numerous previous studies have reported a lack of correlation between preoperative sCEA levels and tCEA expression (8, 28, 29), some literature supports the existence of a relationship (30). Our study demonstrated a positive correlation between sCEA and tCEA. Consequently, we utilised combined CEA as a new variable.

High preoperative s-CEA levels are associated with poor survival outcomes in patients with CRC (31, 32). High-intensity t-CEA expression correlates significantly with increased tumour recurrence rates (33). To further analyse the five-year OS, we performed ROC analysis for sCEA, tCEA, and combined CEA using the MultiVarTimeRoc package with “timeROC.” The results indicated that the AUC for combined CEA surpassed that of sCEA and tCEA alone, highlighting the value of combined CEA as a new factor in assessing five-year OS (Figure 2B). In this study, no significant differences in five-year OS were observed for stages 0 & I and II with respect to sCEA and tCEA, which contrasts with our previous study (8). This discrepancy may be attributed to the larger patient cohort in the current study. Furthermore, there were no differences in five-year OS for combined CEA in stages 0 & I and II. However, in advanced CRC (stages III and IV), significant differences were noted among the three factors, with the exception of tCEA in stage IV. The reason for this is unclear, but it suggests that relying solely on tCEA for prognostic assessment may have limitations. The predictive capacity of tCEA expression intensity for recurrence was particularly pronounced among patients with low preoperative sCEA levels, as those exhibiting high-intensity t-CEA expression showed significantly higher recurrence rates regardless of their low preoperative s-CEA levels (10). When categorised into four subgroups based on both preoperative s-CEA level and t-CEA expression intensity, DFS was poorer in groups with high-intensity t-CEA expression, irrespective of preoperative s-CEA levels. These findings imply that t-CEA expression intensity serves as a complementary measurement to preoperative s-CEA levels (10). In this study, patients with tCEA normal & CEA+ exhibited better five-year OS in stage III but worse five-year OS in stage IV. This intriguing finding may stem from other important prognostic factors in stage IV CRC, such as differentiation, TNM classification, and treatment sensitivity following surgery. In stage III, combined CEA with sCEA normal & tCEA+ exhibited the best prognosis, while sCEA high & tCEA+++ presented the worst prognosis for five-year OS. In stage IV, tCEA showed no significant prognostic value, whereas combined CEA indicated that tCEA high & CEA+++ had the worst five-year OS, while tCEA high & CEA++ had the best five-year OS, suggesting that combined prognostic factors may exert influence at this stage (Figures 5, 6).

Univariate analysis indicated that tumour location, T stage, differentiation, chemotherapy, TNM stage, tCEA, and combined CEA play prognostic roles in CRC, while gender, complications, and sCEA do not. The variables demonstrating significance in univariate analysis were further analysed in a multivariate context, revealing that tumour location, T stage, differentiation, TNM stage, and combined CEA are independent factors in CRC, which aligns with findings from our previous study to some extent (8) and other studies (10, 11). Our previous study indicated that sCEA is not an independent factor, while tCEA is, for CRC stages I–III by multivariate analysis (8). Given the controversial role of preoperative serum markers in CRC, it is recommended to combine preoperative CEA with other tumour biomarkers (17, 19, 34–40). In Figures 5, 6 sCEA, tCEA and combined CEA in stage III, but only sCEA and combined CEA have significant difference while tCEA does not have indicating that only tCEA examination may have defects for CRC prognosis. Using Cox regression analysis, we found sCEA have no significance in univariate analysis, and tCEA do not have significance in multivariate analysis, but combined CEA have both in univariate and multivariate analysis. This study can demonstrated the importance and affects of combined CEA for CRC prognosis. In this study, with a larger patient cohort across stages 0 & I–IV, sCEA and tCEA were identified as prognostic factors but not independent factors in CRC stages III–IV, whereas combined CEA emerged as an independent factor in advanced CRC according to both univariate and multivariate analyses.

## 5 Limitations

This study has several limitations, including the absence of genetic analysis, reliance on outdated data, and the use of preoperative sCEA and tCEA testing kits that are not the most recent (ref. 0-5 ng/mL) which are proformed by the chemiluminescence microsphere immunoassay (ARCHITECT). The use of older ELISA kits and techniques may affect the accuracy of biomarker measurements. We cannot provide a quantitative analysis of tCEA because it is a clinical and relative large data, mainly it is a retrospective study. IHC are widely used to detect tCEA staining in clinical practices. We cannot supplemented the analysis in this paper. Additionally, the determination of t-CEA expression patterns may be somewhat subjective, as these evaluations depend on the depth of CEA distribution. The assessment of tCEA intensity is prone to subjectivity, potentially introducing variability in the results. Furthermore, we did not compare other serum tumour markers, such as carbohydrate antigen 199 (CA199), carbohydrate antigen 724 (CA724), and carbohydrate antigen 125 (CA125), to CEA in this study due to the lack of available data. The study lacks a genetic component or analysis of other tumor biomarkers (e.g., CA19-9, CA724), which could provide a more comprehensive understanding. We do not have these valuable data of sCEA change in follow up period. In future study, we may try to collect sequence data of sCEA in the follow up period, the data is valuable because sCEA may have association with tumor recurrence and metastasis for CRC. We plan to collect these tumour markers in future research. It is also important to note that stage IV refers only to patients with resectable stage IV colorectal carcinoma and does not include all stage IV patients limiting the generalizability of the findings. The study is based on data from a single medical center, which could limit the applicability of the findings to a broader population.

## 6 Conclusion

sCEA, tCEA, and combined CEA serve as prognostic factors in stages III and IV of CRC; however, only combined CEA is identified as an independent factor in these stages, while none of the markers show prognostic relevance in stages 0 & I–II. Combined CEA can be regarded as a new factor for assessing CRC prognosis. Future research may involve integrating postoperative sCEA, recurrence CEA (rCEA), faecal CEA, CA199, CA724, and other tumour biomarkers with tCEA or preoperative sCEA to further investigate their prognostic roles and mitigate the limitations associated with single biomarker testing.

## Data Availability

The original contributions presented in the study are included in the article/supplementary material, further inquiries can be directed to the corresponding author or first author.
